# The Det.Belt Server: A Tool to Visualize and Estimate Amphipathic Solvent Belts around Membrane Proteins

**DOI:** 10.3390/membranes11070459

**Published:** 2021-06-22

**Authors:** Veronica Zampieri, Cécile Hilpert, Mélanie Garnier, Yannick Gestin, Sébastien Delolme, Juliette Martin, Pierre Falson, Guillaume Launay, Vincent Chaptal

**Affiliations:** 1 EMBL Grenoble, 71 Avenue des Martyrs, CS 90181, CEDEX 9, 38042 Grenoble, France; vzampieri@embl.fr; 2 Molecular Microbiology and Structural Biochemistry Laboratory (CNRS UMR 5086), University of Lyon, IBCP, 7 Passage du Vercors, 69367 Lyon, France; cecile.hilpert@ibcp.fr (C.H.); melanie.mc.garnier@gmail.com (M.G.); yannickgestin@gmail.com (Y.G.); sebastien.delolme@ibcp.fr (S.D.); juliette.martin@ibcp.fr (J.M.); pierre.falson@ibcp.fr (P.F.)

**Keywords:** detergent belt, amphipathic solvent, detergent quantification and modeling

## Abstract

Detergents wrap around membrane proteins to form a belt covering the hydrophobic part of the protein serving for membrane insertion and interaction with lipids. The number of detergent monomers forming this belt is usually unknown to investigators, unless dedicated detergent quantification is undertaken, which for many projects is difficult to setup. Yet, having an approximate knowledge of the amount of detergent forming the belt is extremely useful, to better grasp the protein of interest in interaction with its direct environment rather than picturing the membrane protein “naked”. We created the Det.Belt server to dress up membrane proteins and represent in 3D the bulk made by detergent molecules wrapping in a belt. Many detergents are included in a database, allowing investigators to screen in silico the effect of different detergents around their membrane protein. The input number of detergents is changeable with fast recomputation of the belt for interactive usage. Metrics representing the belt are readily available together with scripts to render quality 3D images for publication. The Det.Belt server is a tool for biochemists to better grasp their sample.

## 1. Introduction

Membrane proteins have, by definition, a part of their structure embedded within the membrane. This part matches the hydrophobic environment of the lipid tail, and therefore displays on the outside a shell of hydrophobic residues. Once extracted from the membrane for biochemical or biophysical studies, this hydrophobic part has to be shielded by amphipathic compounds. Failure to correctly protect this region undoubtedly results in protein aggregation or loss against any hydrophobic surface of a test tube. Many types of amphipathic compounds are available today to perform this task [1,2]. Detergents are popular tools and exist in different varieties, varying from the length and shape of aliphatic side chains to different hydrophilic moieties. These differences give rise to distinct properties and behaviors around membrane proteins, and new detergents are being created to enhance stability or activity of target membrane proteins [3,4,5,6,7]. Aside from detergents, several types of polymers have also been developed to wrap around the hydrophobic region, directly at the solubilization stage or after purification in detergents [8,9,10,11,12].

For many years, these amphipathic compounds have stayed in the shadow of membrane proteins, being the ghosts that are always present but seldom seen, despite the huge influence on the membrane protein function. Methods to quantify these amphipathic compounds have stayed limited to specific detergents or difficult to setup [13]; the reference method being the use of radioactivity [14,15] was inherently limited by the need to synthetize dedicated radioactive detergents. Recently, a new method was released based on MALDI-ToF mass spectrometry, to quantify any detergent in a variety of settings, presence of lipids or complex mixtures, rendering the detergent quantity readily available for any membrane protein around virtually any detergent [16].

Aside from quantification, detergent visualization was achievable by only a few methods. Pioneer work on neutron diffraction of membrane protein crystals with deuterated detergents revealed the arrangement of detergents in a belt wrapping around the membrane part of proteins [17]. Later, detergent belts were also observed by cryoEM, confirming the similar type of architecture around the membrane part of proteins [18,19]. It is important to note that in cryoEM and the method of single particle analysis (SPA), particle alignment is critical in enhancing the signal of particles in each orientation. However, the detergent belt is very mobile as was shown by molecular dynamics simulations of detergents [16]. The averaging process of SPA thus truncates a part of the detergent signal, resulting in belts smaller than they really are [20].

In addition to the exact representation of the belt surrounding the hydrophobic region, it is just as essential to have a good understanding of the general amount of compounds forming the belt. Too often it can be found in the literature that a detergent micelle is surrounding the hydrophobic region. In fact, micelles are completely separate objects as the belt around membrane proteins. As an example, Dodecylmaltopyranoside (DDM), one of the most used detergents [2], has an aggregation number of around 80–100 monomers to form a micelle. In contrast, 400 monomers have been quantified to form a belt around ABC transporters [16,21] and about 200 around the human purinergic receptor [16]. While these numbers are interesting in themselves, it is even more useful to visualize their contribution around the given membrane protein. We thus created the Det.Belt server, which provides a tool for biochemists to determine the amount of amphipathic compounds surrounding the membrane protein of interest. This tool is available to the community as a web server: https://www.detbelt.ibcp.fr (manuscript version released 24 March 2021).

Here we report the creation of the Det.Belt server to represent detergent belts around membrane proteins. This server is a tool to evaluate the gross amount of detergent that surrounds any membrane protein for which a PDB code can be provided, and can be used to predict the amount of detergent around a new target. The amount of detergent estimated using Det.Belt is useful for example in reconstitution assays where knowing the amount of detergent is key to estimate liposome swell and detergent saturation values.

## 2. Materials and Methods

### 2.1. Server Input

The server takes as input a 3D structure in the PDB format. The structure must be positioned in the lipid membrane by the PPM server [22,23]. Alternatively, a pre-oriented membrane protein database can be queried by keywords or PDB codes. This database was created by combining information coming from two databases of membrane proteins. The first resource is the XML release of the Membrane Protein Topology (MPTOPO) Database (https://blanco.biomol.uci.edu/mptopo/) (accessed on 15 December 2020), which provides a detailed description and classification of membrane proteins. The second resource is the Orientations of Proteins in Membranes (OPM) database (http://opm.phar.umich.edu) (accessed on 15 December 2020), which provides a large collection of membrane protein structures positioned in a lipid bilayer. The PDB structure files initially fetched from the OPM database were sorted and annotated according to the classification of the MPTOPO database. Structures are thus oriented with the z axis normal to the membrane plane and coordinate origins at the center of the membrane.

The server also takes as input the number of detergent molecules around the protein. Several detergent types can be selected. The number of detergent molecules is used to compute the total volume of detergent, using nominal detergent volumes obtained from the program VOIDOO [24]. SMILES string for each detergents were retrieved from 2D chemical structures using the NIH web server (https://cactus.nci.nih.gov/translate/index.html) (accessed on 15 December 2020) and 3D structures were created and energy minimized using the program Elbow in PHENIX [25].

### 2.2. Calculation of the Belt Dimensions

The detergent is represented by a belt, formally a hollow cylinder, whose dimensions are computed as follows. The belt height, h, is read directly from the input structure. PDB files processed by the PPM server typically contain a REMARK line with the value corresponding to half of the membrane thickness. We simply multiply this value by two to get the belt height. The inner radius of the belt, *r*, is computed from the transmembrane part coordinates. We first compute accessible surface area of all heavy atoms of the input structure by using NACCESS [26]. Atoms of the transmembrane part (deduced from the z coordinates) with a solvent accessible surface area greater than 3 Å^2^ are kept aside and their radial distance to the z axis is computed. From the distribution of the radial distances, we extract the value corresponding to the rightmost peak in the density. The inner radius is equal to this value with 1.66 Å added to account for the average van der Waals radius of heavy atoms. The outer radius of the belt, *R*, is deduced from the belt height, *h*, and belt inner radius, *r*, such as to generate a hollow cylinder with a volume equal to the total volume of detergent, *V*:

$$R=\sqrt{\frac{V}{\pi h}+R^{2}}$$

### 2.3. Server Implementation

The protein of interest can be interactively visualized in 3D, both prior to submission and after the computation of its solvent belt. The 3D representation of the protein is carried by the NGL library, while solvent volumes are drawn with the ThreeJS library (https://threejs.org/) (accessed on 15 December 2020). The computation of the detergent belt is performed by a set of Python and R scripts freely available at https://github.com/MMSB-MOBI/detbelt_coreScripts (accessed on 20 June 2021).

All of the metrics described above (*h*, *r*, *R*, *V*) are available on display and for download. A Pymol [27] script is also available for download.

## 3. Results

### 3.1. Det.Belt Web Interface

**Figure 1:**
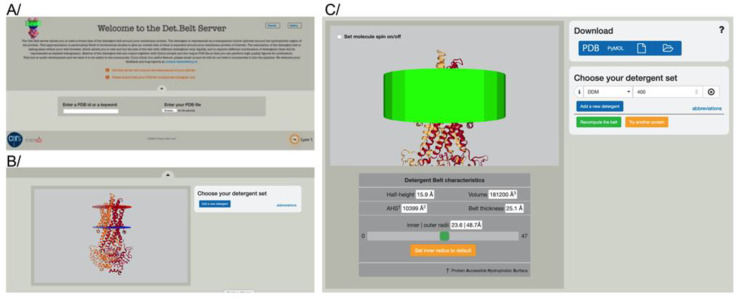
Det.Belt web interface. (**A**) Home page where investigators can either search for a pre-oriented membrane protein structure using PDB identifiers or keywords, or provide their own oriented structure file. (**B**) Display of the oriented protein. The red and blue spheres represent the extracellular and cytoplasmic sides of the membrane, respectively. Each chain is shown as a cartoon in a different color. (**C**) Typical output from Det.Belt shown here with the DDM belt displayed in green. PDB code used in this figure: 2hyd.

In order to represent the detergent belt around a membrane protein, a structure file coming from the Protein Data Bank [28] or a typical refinement or model building software must be provided. The membrane insertion of the protein is used for all downstream calculations, which is provided by the “Orientation of Protein in the Membrane” server [22]. Investigators can query a pre-processed local database, where we downloaded the structure files for integral membrane proteins, and pre-oriented them. Investigators can search for either the PDB identifier or keywords relevant to their proteins (Figure 1A). Users can also upload their own PDB file, which is required to be pre-oriented by the “Orientation of Protein in the Membrane” server [22]. The protein is represented in cartoon with a color per monomer. The red and blue spheres correspond to the membrane insertion; the blue spheres correspond to the cytoplasmic side (Figure 1B).

The membrane insertion for each protein is then approximated as a cylinder. The volume of the belt corresponds to the amount of detergent input by the user multiplied by the volume occupied by each detergent and is represented as a hollow cylinder (Figure 1C). A color was assigned to each class of detergent to distinguish them easily. The result is displayed using OpenGL and is interactive; users can then move and zoom on their protein in the web browser window and check the result of the data submitted. Metrics are available to users for downstream processes in the window below the graphic representation. A Pymol [27] script is downloadable to create high resolution figures, and the metrics are generated in the download tab (Figure 1C).

The number of input detergents is changeable, triggering an immediate updated representation and new metrics upon a belt recomputation request. The type of detergent can also be modified to allow investigators to have a feel of what their protein would look like when surrounded by distinct types of amphipathic molecules.

A gallery page is available with examples of curated data where detergents have been formally quantified [16] for reference and comparison (Figure 1A).

### 3.2. Detergent Belt Representation as a Hollow Cylinder

It has been previously shown by neutron diffraction of membrane proteins crystals that detergents wrap around membrane proteins to form a belt shielding the hydrophobic part defining the membrane insertion [17,29,30]. This belt is shaped as a taurus. The one observed by cryoEM roughly matches the same taurus [20]. Both methods use an averaging procedure that results in seeing the commonality between proteins within the crystal or particles on the grid, respectively, thereby removing differences between individual proteins/particles. Molecular dynamics simulations of detergents show, however, a very dynamic belt with a very fluid phase made from detergents, which deforms locally and dynamically [16,31,32,33]. With these in mind, we decided to represent the detergent belt as a hollow cylinder for several reasons. First, it is very fast to calculate and is thus compatible with an online server. Second, for the reasons invoked above, representing the belt as a taurus would represent an equally biased representation. The main focus of this server is to give investigators a view of how much/many detergents are present and to give them a new view of their sample. The hollow cylinder representation is obviously nonphysiological and would thus trigger critical thinking about the output, while at the same time providing a fair estimate of the real detergent distribution.

### 3.3. Grasping the Correct Amount of Detergent around Any Membrane Protein

**Figure 2:**
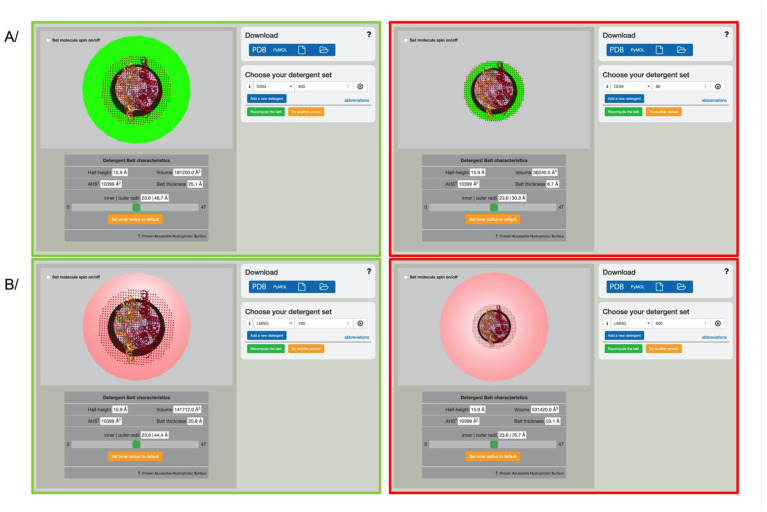
Interactive detergent belt visualization. (**A**) ABC transporter with a DDM belt modeled (green belt). The left panel shows the measured amount of DDM (400 monomers), while the right panel shows the same protein embedded in a DDM micelle (80 monomers). (**B**) same as (**A**) in the detergent LMNG (pink belt). The left panel displays the 160 monomers measured, while the right panel shows an arbitrary micelle number of 600. PDB code used in this figure: 2hyd.

The detergent “micelle” surrounding membrane proteins is often referred to. Once visualized around the protein, it becomes immediately apparent that this phrase does not correspond to the detergent belt surrounding membrane proteins. An example is given for two popular detergents, DDM and LMNG. A DDM micelle is made of 80–100 monomers (aggregation number), depending on the measurements. A LMNG micelle is very different from the DDM one, measured by SAXS/SANS to be an elongated rod varying in size, and with aggregation numbers varying from >200 to 600 [16,34]. For comparison, the measured amount of detergents forming the belt around an ABC transporter is 400 DDM and 80–150 LMNG (depending on the method used for LMNG) [16,34]. Figure 2 shows several belts obtained by using the “recompute belt” button, applied around an ABC transporter. The measured number is displayed on the left. It is immediately apparent that the number of DDM present in a micelle is too small to shield the hydrophobic surface area of this transporter. In contrast, the LMNG micelle aggregates far too many monomers to provide a simple protection of the hydrophobic surface area. Far from the micelle properties of detergents, which have their own intrinsic rules, membrane proteins gather the right amount of detergent to shield their hydrophobic region; this amount differs from detergent to detergent, and is unrelated to their micelle properties [15,16,34,35]. Following this idea, investigators can freely explore the size given by a different number of detergent and detergent-types, thereby gaining some knowledge of how many monomers would be needed to cover the hydrophobic surface area of their protein of interest.

### 3.4. Detergent Mixtures

**Figure 3:**
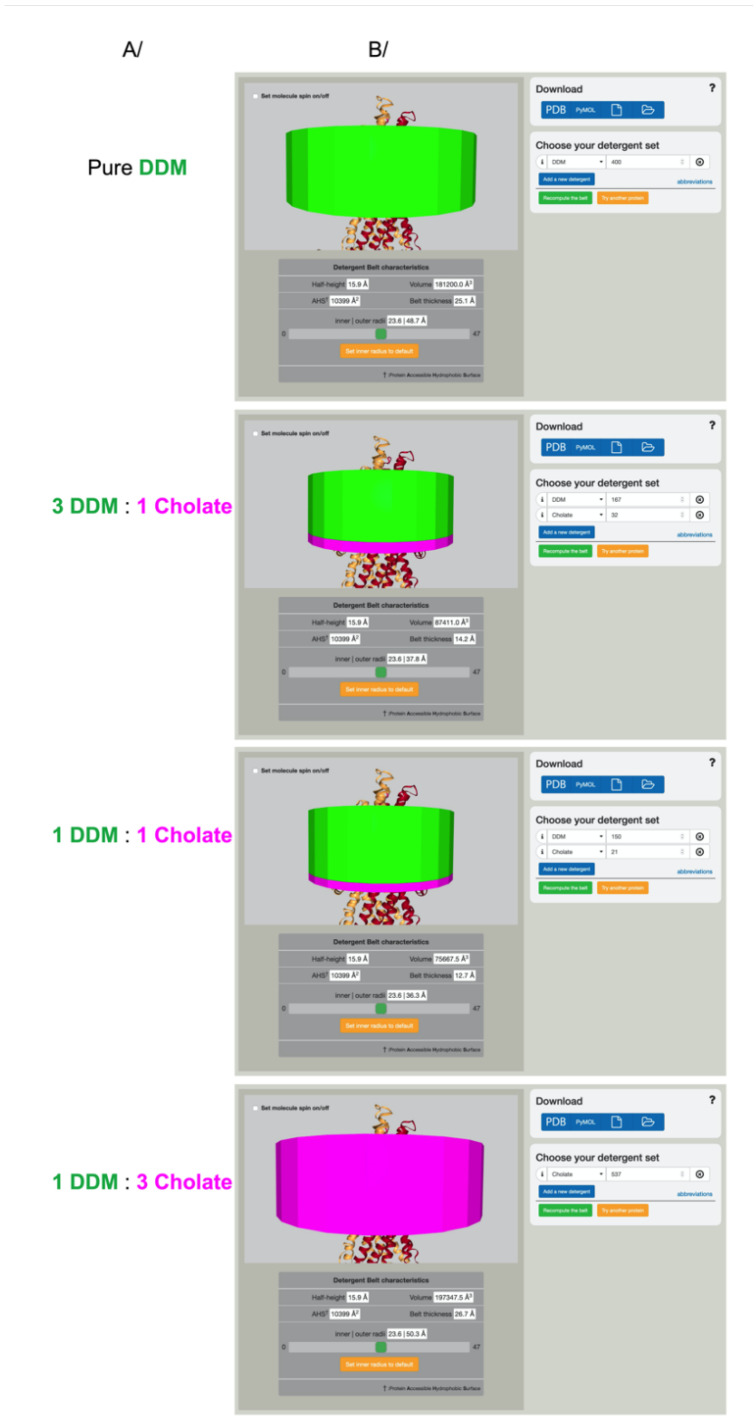
Representation of detergent mixtures. (**A**) Purification condition for the ABC transporter BmrA as described in [36]. The ratios define the detergent’s molar ratio used during the purification. (**B**) The measured number of detergents is depicted using Det.Belt. Note that the measured amounts differ completely from the detergent ratio in purification buffers [36]. PDB code used in this figure: 2hyd.

Detergents are often used in mixtures, and it is possible to represent several detergents forming the belt, each having their own number of monomers. We must emphasize that detergent mixtures do not behave as single detergents, and also do not behave around the protein as they behave in mixed micelles [36]. Very few data are available on detergent mixture quantification; an example of the mixture DDM and cholate has been extensively described in [36] (Figure 3). We chose to represent detergent mixtures as stacked cylinders, with each stack representing the volume occupied by the input number of each detergent. Colors are given for each type of detergent. The stacking of detergents is not physiological, it is a representation of the volume occupied by each detergent. For a more physiological representation of the detergent mixture, investigators are encouraged to explore this behavior using molecular dynamics simulations.

### 3.5. Application to Detergent Prediction

The amount of detergent embarked around a membrane protein depends on the nature of the membrane protein (titled trans-membrane domain or straight) and of the hydrophobic surface area to cover [15,16]. Using the gallery page of the Det.Belt server, investigators acquire an idea of precisely measured detergent amount in various purification settings. Using this, investigators can easily extrapolate the amount of detergents embarked around their membrane protein of interest. Precise quantification is not always required for most applications, and often a rough idea is already very instructive to make informed choices on samples and sample downstream processing. An example is membrane protein reconstitution into liposomes. Very often, this method requires in-depth and controlled knowledge of the liposome swell once detergents are added to the mixture, in order to not solubilize the liposome but merely to destabilize it and allow protein reconstitution [37,38,39,40]. Using the Det.Belt server, investigators can make an informed guess on the amount of detergent present in their sample. This will narrow the search for optimal reconstitution conditions. It also allows the use of the table in Rigaud et al. to determine the amount of Biobeads needed to remove the detergent of their sample [41].

### 3.6. Detergent and Lipids Database

**Figure 4:**
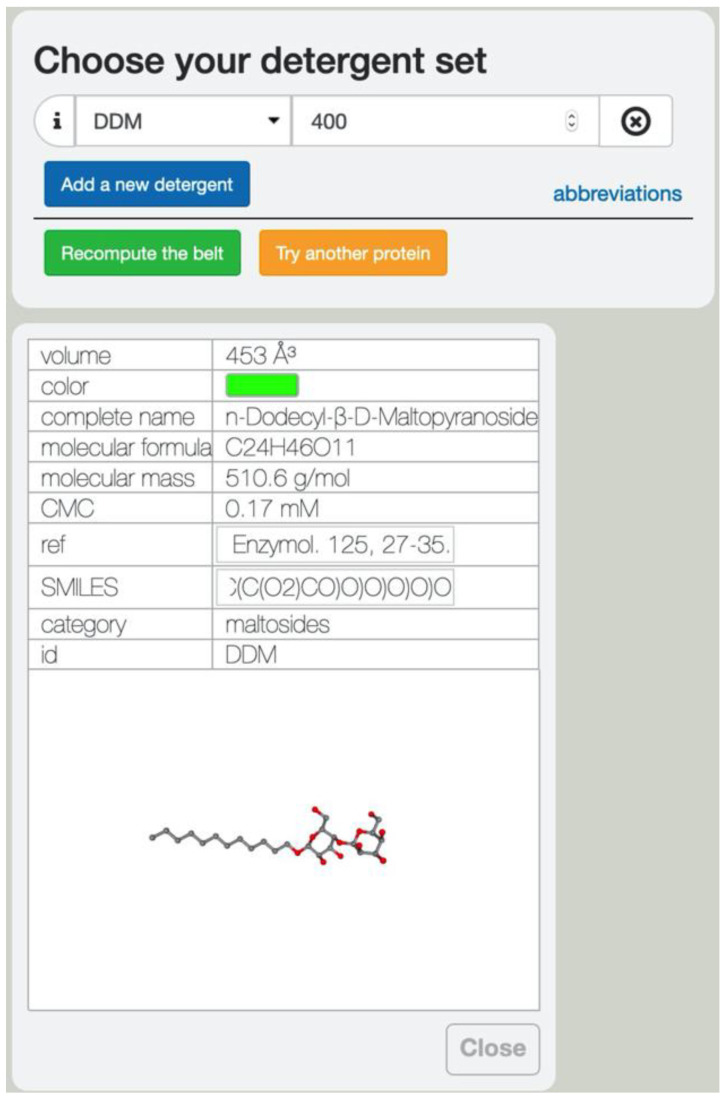
Detergent database. All of the information relative to each entry in the database can be obtained by clicking on the “i” next to the detergent name. The SMILES string that was used to build the molecule is available and the detergent molecule is displayed in 3D in the lower window.

All of the detergents and lipids displayable in Det.Belt were assembled into a database. For each entry, the database contains the full name and chemical formula, the molecular weight, the critical micellar concentration, the reference from where these information were derived, the calculated volume, and the SMILES string, so that it is easy to retrieve structural information for downstream studies. This information appears in a box once the detergent has been selected by the user, with a 3D view of its chemical structure (Figure 4). If the investigator asks to visualize several detergents, a tab appears for each detergent queried. The database includes detergents from the maltoside, glucoside, neopentyl glycol, cholesterol derivative, fos-choline, and amine oxide families, as well as thio-derivatives of some of these detergents. Some common lipids are also included, as well as the polymer amphipol, which is commercially available (A8–35, [42]). Detergents are abbreviated using their common name, and a list of abbreviations is also provided.

### 3.7. Special Cases

There are cases where the membrane protein structure cannot be strictly approximated as a cylinder. Typical examples are oligomers that can show elongated or spherical forms but display deep grooves in between monomers. Large complexes also fall into this category. For these cases, the hollow cylinder representation is a suboptimal fit, but nevertheless allows for understanding the quantity of detergents surrounding the protein.

**Figure 5:**
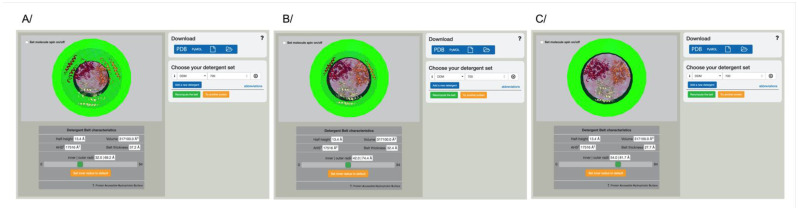
Variation of the detergent belt by changing the inner radius. The scroll bar can be adjusted to displace the position of the inner radius for the detergent belt cylinder, larger or smaller. Metrics are automatically adjusted, as well as the display. The belt can also be set back to the original default value.

We tried to implement a measure that could be an average of long and small distances for the membrane protein cylinder description, but failed to find a solution that would fit all the proteins due to their variability. We thus implemented a scroll bar for investigators to choose and fine tune the definition of the hollow cylinder that would best fit the protein of interest (Figure 5). When the size of the inner cylinder is changed, all the metrics are immediately recalculated and the display updated. Figure 5B shows the server output for the multidrug transporter AcrB surrounded by 700 DDM monomers. The default representation shows part of the AcrB trimmer sticking into the green cylinder, while other parts in-between trimmers are facing an apparent vacuum. It is possible to decrease the size of the inner cylinder (Figure 5A) to include the parts that were previously naked, or inversely, increase the radius to show the belt in the outmost position (Figure 5C), all with the amount of detergent unchanged. In reality, no part of the structure will be embedded in detergent or left naked, and these are small artifacts from the representation. Still, using this representation, it is possible for investigators to estimate how many detergent monomers are around the protein of interest.

## 4. Discussion

Detergents are ghosts to biochemical studies of membrane proteins, always present but seldom seen. Investigators know how much they put into buffers and all the purification steps, but do not have immediate feedback on what is left around the membrane protein. Additionally, detergents can have side effects, varying from mild loss of activity to complete denaturation in extreme cases [1,3,43,44,45]. Since the effect of detergents is hard to grasp, false impression can originate and lead to misleading data interpretation. The Det.Belt server is a tool that was created to rationalize detergent use by giving investigators an idea of how many detergents are riding along with their protein. Using this server, it is possible to vary the number of detergent monomers, and the type of detergent, thereby virtually screening within seconds hundreds of conditions and visualizing the belt around any membrane protein. The use and interest of the Det.Belt server is clearly summarized in the sentences above, to bring awareness of what is occurring in the test tube, since investigators do not have a means to directly observe it.

It is important to stress that the actual real behavior of amphipathic compounds around membrane protein is influenced by many external factors. The first and most important behavior to acknowledge is the dynamic characteristic of detergent belts, as observed in molecular dynamics simulations [16,46,47]. Local deformations of the belt are constantly happening with time, and seen at any part of the belt. This results in a rough-edged belt if observed from up-close, but that could also be seen as somewhat smooth if viewed from a fair distance and over time. This guided the choice of a hollow cylinder to render the volume occupied by the detergent belt. This cylinder is at the same time unphysiological in its shape, and a good estimate of what actually occurs. This representation has been chosen to trigger critical thinking of detergent belts for users of the Det.Belt server.

Similarly, we opted to render detergent mixtures as stacked cylinders showing the portion of the volume occupied by each detergent with the idea to stress caution in the interpretation of the data. Indeed, belts consisting of detergent mixtures have rarely been deeply investigated, and yielded many surprises when thoroughly quantified [36]. The server is thus not rendering detergent as belts radiating away from the protein, as this can lead to misinterpretation on the positioning of each detergent within the mixture. For a better description, molecular dynamics is the only tool that can bring an accurate description, and investigators are encouraged to pursue this avenue to continuing future studies.

Detergent quantification is the only way to know the characteristics of the sample, but could be a deleterious task for many laboratories and requires specific equipment. Using previous quantification as a base, the Det.Belt server alleviates the need to quantify detergents for each protein, while giving investigators a good idea of where their sample is standing in respect to their experiment. The Det.Belt server is thus a tool for structural biochemists to better understand their sample.

## Data Availability

The data presented in this study are available on request from the corresponding author.
